# Silent progressive bilateral papillitis after COVID-19 vaccination: A case report

**DOI:** 10.1097/MD.0000000000031112

**Published:** 2022-10-14

**Authors:** Geun Woo Lee, Hyeong Seok Park, Donghun Lee

**Affiliations:** a Department of Ophthalmology, Daegu Catholic University School of Medicine, Daegu, Korea.

**Keywords:** bilateral papillitis, case report, coronavirus disease 2019, optic neuritis, vaccination

## Abstract

We report the case of a patient who developed asymptomatic bilateral papillitis after coronavirus disease 2019 (COVID-19) vaccination.

**Patient concerns:**

A 61-year-old man presented to our tertiary clinic with bilateral optic disc edema, which was incidentally detected during his visit to a primary ophthalmology clinic. He had received an adenovirus-vectored COVID-19 vaccine 2 weeks before the optic disc edema was detected and had experienced no ocular discomfort, except for a floater in his right eye. Although his visual acuity was normal and he had no color vision deficiencies or marked visual field defects, the optic disc edema worsened over several days. Orbital magnetic resonance imaging showed no optic tract enhancement, and lumbar puncture revealed normal cerebrospinal fluid pressure. The patient tested negative for aquaporin-4 and myelin oligodendrocyte glycoprotein antibodies and Leber hereditary optic neuropathy-associated gene mutations.

**Diagnosis:**

The patient was diagnosed with bilateral papillitis, possibly induced by the COVID-19 vaccination.

**Interventions:**

The patient received steroid pulse therapy with methylprednisolone (500 mg/day) for 3 days followed by an oral prednisolone taper for 3 weeks.

**Outcomes:**

The patient’s papillitis started to subside 3 weeks after he received systemic steroid therapy and completely resolved without any sequelae 2 months later. A year after the diagnosis, the fundus remained stable without disease recurrence or optic disc atrophy.

**Lessons:**

Healthy individuals receiving COVID-19 vaccines may present with various manifestations of optic neuritis. In the present case, the patient presented with asymptomatic progressive bilateral optic disc edema and had a favorable long-term course after receiving steroid therapy.

## 1. Introduction

Since the coronavirus disease 2019 (COVID-19) vaccine was first approved for use in Western countries in December 2020,^[1]^ various neuro-ophthalmic manifestations associated with COVID-19 vaccination have been reported to be comparable to those associated with the COVID-19 infection itself in patients. Neuro-ophthalmic complications include eye disorders such as neuroretinitis, optic neuritis, nonarteritic anterior ischemic optic neuropathy, and retinal vessel occlusion^[2]^ and eye disorders concomitant with brain lesions such as paralytic strabismus and papilledema after thrombotic events or autoimmunological inflammation.^[3–5]^

In most of the reported studies, patients presented with prominent visual dysfunctions such as decreased visual acuity, diplopia, and visual field defects,^[3,6,7]^ and the causes of these disorders were confirmed; lesion localization in brain imaging or positive biomarkers in laboratory test.^[4,8,9]^ However, a case with asymptomatic bilateral optic disc edema, which was followed for a long period of one year is rare. Herein, we present the case of a patient who developed silent progressive bilateral papillitis after receiving an adenovirus-vectored COVID-19 vaccine. The patient has provided informed consent for the publication of the case.

## 2. Case report

A 61-year-old man without any systemic diseases presented to our hospital with a floater in his right eye, which had appeared 2 days prior to presentation. He was well-being appearance without eye pain or headache. He had a history for receiving his second dose of an adenovirus-vectored COVID-19 vaccine (ChAdOx1-S, Vaxzevria, AstraZeneca, Cambridge, UK) 2 weeks before presentation. Five days after vaccination, he was admitted to a different clinic owing to fever of unknown origin, and he recovered with conservative treatment. He reported that the floater appeared after the fever subsided.

**Figure 1.**
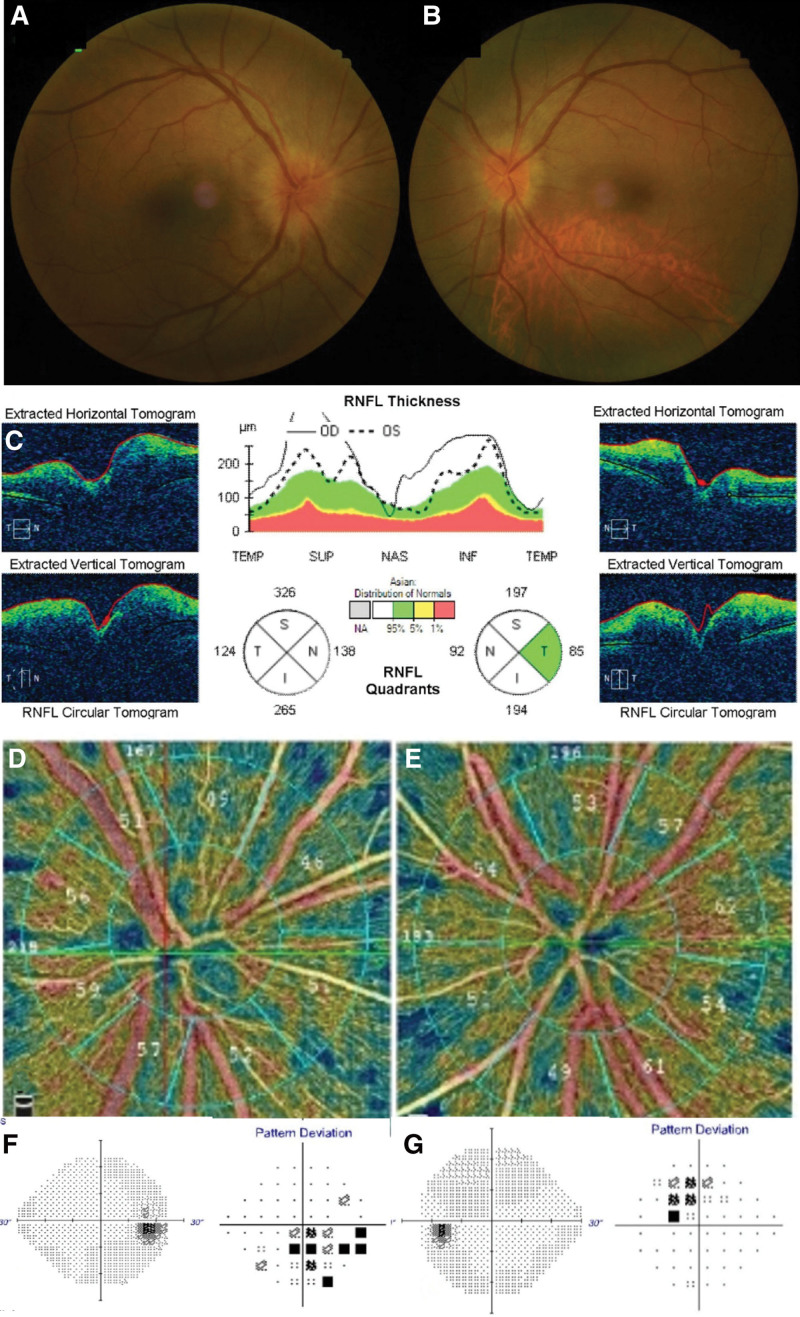
Ophthalmic examination at initial visit. Funduscopy revealed optic disc edema in both eyes, especially severe in the right eye (A) comparing to the left eye (B). On the optical coherence tomography (OCT), average peripapillary retinal nerve fiber layer (RNFL) thickness was increased in both eyes (C). There was no significant ischemia on the right (D) and left (E) disc area on the OCT angiography. In the visual field test, mild field defect in the lower side including the central area in the right eye (F) and minimal superior and central scotoma in the left eye (G) was noted.

On his first visit to our hospital, he had a best-corrected visual acuity of 20/20 in both eyes and normal intraocular pressure. His Ishihara test results for color perception were normal and extraocular muscle movements were full. Pupillary light reflex examination showed that his pupils were equal and reactive with no afferent pupillary defect. Slit-lamp examination revealed no cells in the anterior chamber or vitreous. Fundus examination revealed bilateral optic disc edema, which was more severe in the right eye (Fig. 1A) comparing to the left eye (Fig. 1B). Optical coherence tomography (OCT) showed that the average peripapillary retinal nerve fiber layer (RNFL) thickness had increased to 213 µm in the right eye and 142 µm in the left eye (Fig. 1C). Optical coherence tomography angiography was used to evaluate vascularity of the optic disc and revealed no ischemia on disc area (Fig. 1D–E). Humphrey visual field testing showed bilateral mild central scotoma. In the visual field test, mild field defect in the lower side including the central area in the right eye (Fig. 1F) and minimal superior and central scotoma in the left eye (Fig. 1G) was noted. Brain magnetic resonance imaging (MRI) revealed no optic nerve inflammation and a normal parenchyma. Laboratory tests including complete blood count, comprehensive metabolic panel, urine analysis, chest X-ray yielded negative or unremarkable results. The C-reactive protein level was 0.10 mg/dL, and the erythrocyte sedimentation rate was 2 mm/h.

**Figure 2.**
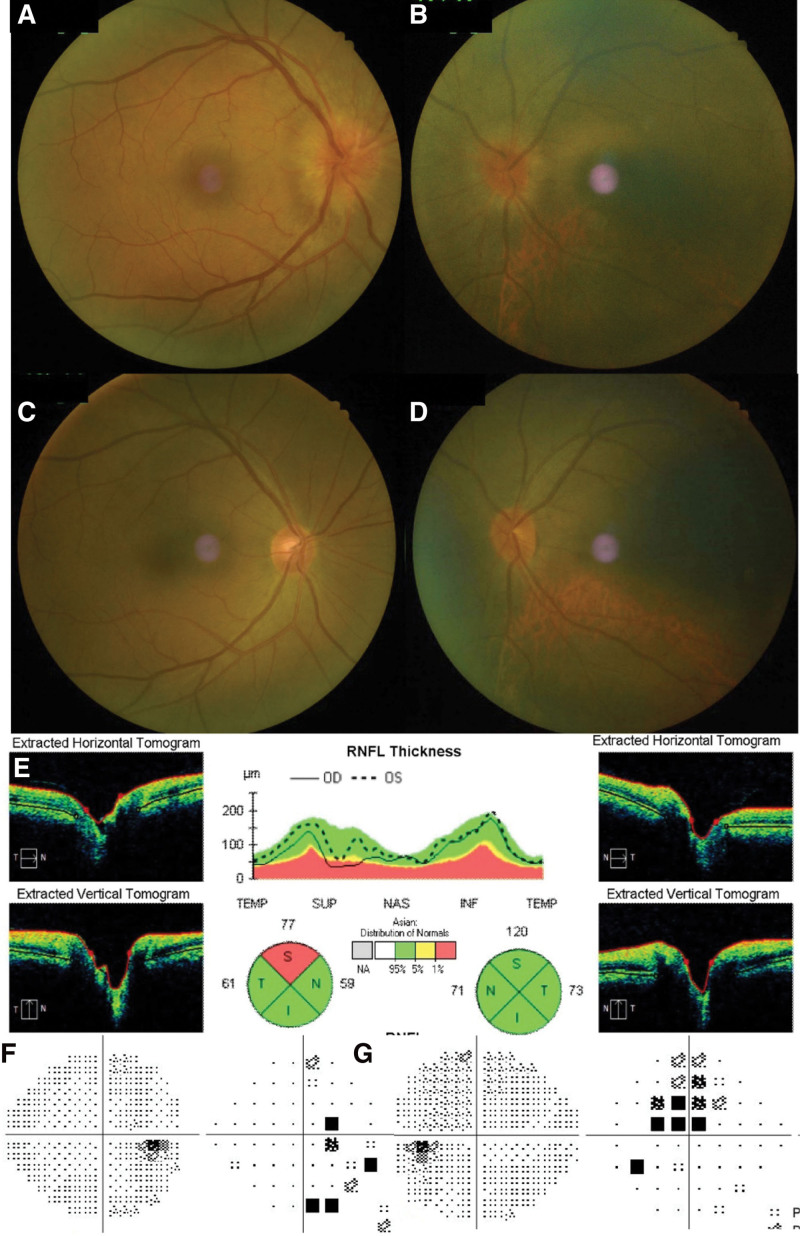
Ophthalmic examination at second visit and after steroid treatment. Fundus examination showed that the disc edema had increased at second visit. And the degree of edema was more severe in the right eye (A) than in the left eye (B). Both optic disc edemas were completely subsided after 2 month (C–D). (C) describes the right eye and (D) is the picture of the left eye. A year after the diagnosis, no further RNFL thinning was detected on OCT (E). There were no significant findings on visual field examination of the right eye (F) and only minimal central scotoma was remained on his left visual field (G). OCT = optical coherence tomography. RNFL = retinal nerve fiber layer.

Under the impression of papilledema, considering Considering that visual function was maintained, the which did not correspond with the severe disc edema, spinal tapping was therefore recommended for measuring cerebrospinal fluid pressure, but the patient refused to undergo the examination. Therefore, regular follow-up without treatment was performed at the clinic. After 2 days, the patient reported that the floater in his right eye had spontaneously disappeared and he experienced no further visual discomfort. However, fundus examination showed that the disc edema had increased, which did not correspond with the reported symptom relief. And the degree of edema was more severe in the right eye (Fig. 2A) than in the left eye (Fig. 2B). For further evaluation, orbital MRI and a spinal tap were performed. Orbital MRI showed no optic tract enhancement or intracranial abnormalities such as ventricular enlargement and mass lesions. The spinal tapping showed normal white blood cell count and biochemical profile results including a normal opening pressure of 120 mmH_2_O. There was no evidence of infection and inflammation in blood test. Screening for autoimmune optic neuritis including testing for anti-myelin oligodendrocyte glycoprotein and aquaporin-4 antibodies and analysis of mitochondrial DNA mutations associated with Leber hereditary optic neuropathy yielded unremarkable results. Polymerase chain reaction test results of the patient were negative for COVID-19.

Considering the clinical symptoms and ophthalmic examination findings of the patient, asymptomatic papillitis was suspected, and high-dose intravenous steroid treatment was initiated. The patient received intravenous pulse methylprednisolone therapy (500 mg/day) with glycemic monitoring for 3 days followed by an oral prednisolone taper for 3 weeks. During the treatment period, the disc edema was consistent and did not improve. Mild enlargement of the physiological blind spot in his right eye was noted in the visual field test, but his visual acuity and color perception remained normal. Three weeks after his first visit to our hospital, the disc edema started to subside, and the peripapillary RNFL thickness decreased. Two months later, superior peripapillary RNFL thinning was observed on optical coherence tomography, but optic disc atrophy in both the right (Fig. 2C) and the left eye (Fig. 2D) was not observed on fundus examination. At the last follow-up, a year after the diagnosis, no further RNFL thinning was detected (Fig. 2E). There were no significant findings on visual field examination of the right eye (Fig. 2F) and only minimal central scotoma was remained on his left visual field (Fig. 2G).

## 3. Discussion

Studies on optic neuropathy associated with COVID-19 vaccination have reported various visual outcomes from visual function improvement in some patients to permanent visual loss in others. In the present study, the patient reported few deficits in visual function at the first presentation, and disc edema was detected only on fundus examination.

Considering that the patient had normal visual function despite severe bilateral disc edema, he was initially suspected to have papilledema or idiopathic intracranial hypertension.^[10]^ However, because his cerebrospinal fluid opening pressure was normal, papilledema and idiopathic intracranial hypertension were no longer considered. Moreover, nonarteritic anterior ischemic optic neuropathy, which is the most common acute optic neuropathy in the elderly population,^[11]^ was ruled out because no ischemic lesions in the peripapillary region and no definite altitudinal field defects were found on optical coherence tomography angiography^[12]^ and visual field, respectively. Furthermore, because there was no infection evidence in blood test and no enhancement in orbital MRI, the patient was finally diagnosed with bilateral papillitis, which was regarded as an exclusion diagnosis.

There have been several case reports that have described bilateral disc edema after COVID-19 vaccination.^[13–15]^ Roy et al^[15]^ reported the case of a 40-year-old man who showed symptoms typical of acute bilateral optic neuritis 12 days after receiving a COVID-19 recombinant messenger RNA vaccine. He experienced vision loss and pain with eye movement, and orbital MRI demonstrated optic nerve enhancement. His best-corrected visual acuity improved after steroid therapy. Helmchen et al^[14]^ reported a case in which a patient with multiple sclerosis developed neuromyelitis optica, an autoimmune disease that distinctly differs from multiple sclerosis, after receiving a vector-based vaccine. The presumptive mechanisms of neuro-ophthalmologic complications associated with COVID-19 vaccination do not differ from those associated with other vaccines.^[16]^ Possible mechanisms include infection with the attenuated pathogen, molecular mimicry resulting in hypersensitivity, and adjuvant-induced inflammation.^[7]^ Furthermore, these are believed to be associated with immune-mediated damage, resulting in demyelination or localized vasculitis.^[17]^ In the present study, bilateral papillitis may have developed owing to an immune response or hypersensitivity in the patient. Moreover, after receiving the vaccine, the patient developed fever of unknown origin, which may have been an adenovirus vector-induced immune response. This may have caused an inflammatory reaction in the optic nerve.

Meanwhile, regarding thrombotic events specific to adenovirus-vectored COVID-19 vaccines,^[18]^ previous studies have reported papilledema development associated with thrombosis in patients after vaccination.^[4,19]^ Qian et al^[19]^ reported the case of a 45-year-old woman who was diagnosed with papilledema secondary to dural sinus thrombosis 8 days after vaccination. Nowak et al^[4]^ reported that a 23-year-old man developed papilledema associated with intracerebral hemorrhage secondary to venous sinus thrombosis 10 days after vaccination. In the present study, the possibility of a microthromboembolic event associated with the adenovirus-vectored vaccine cannot be excluded; however, unlike that reported in the previous studies, our patient did not experience systemic symptoms such as headache or dyspnea that can occur owing to thrombosis in other organs.

Regarding the patient’s response to steroid therapy, the reasons for why the patient’s disc edema did not decrease for several weeks after steroid therapy and then slowly subsided thereafter are unclear. We suggest several hypotheses to explain this result. First, the patient’s signs and symptoms were not severe, which may mean that his immune response to COVID-19 vaccination was not strong. Therefore, his responses to steroid therapy for suppressing abnormal immune reactions may have been also slow and not prominent. The second hypothesis is associated with the steroid dosage. Considering his old age and minimal visual function impairment, the patient received therapy with half the standard steroid dose reported in the Optic Neuritis Treatment Trial.^[20]^ If he had received the conventional high-dose steroid treatment, he could have shown a dramatic response to the steroids.

Although we have reported only one case of bilateral papillitis associated with COVID-19 vaccination, we believe that more data on the development and characteristics of optic neuritis in patients receiving different types of COVID-19 vaccines would be meaningful.

## 4. Conclusion

In the present study, we report the results of a 1-year follow-up in a patient who developed asymptomatic bilateral disc edema after COVID-19 vaccination. Contrast-enhanced orbital and brain MRI showed unremarkable results. The results of systemic evaluations including spinal tapping and laboratory tests did not support the possibility of papilledema, infection, or other autoimmune diseases.

In addition, because the patient was healthy without any underlying systemic diseases and his disc edema was detected 2 weeks after vaccination, he was diagnosed with bilateral papillitis associated with COVID-19 vaccination. After high-dose intravenous pulse steroid treatment, the disc edema gradually subsided. A year after the diagnosis, the patient’s fundus remained stable without visual sequelae or disease recurrence. This case report demonstrates that atypical asymptomatic papillitis can occur after COVID-19 vaccination.

## Author contributions

**Conceptualization:** Donghun Lee.

**Data curation:** Hyeong Seok Park, Donghun Lee.

**Investigation:** Geun Woo Lee, Donghun Lee.

**Resources:** Hyeong Seok Park, Donghun Lee.

**Writing – original draft:** Hyeong Seok Park, Donghun Lee.

**Writing – review & editing:** Donghun Lee.
